# Intravesical recurrence of chromophobe renal cell carcinoma

**DOI:** 10.1016/j.eucr.2023.102479

**Published:** 2023-06-29

**Authors:** Niall P. Kelly, Liza McLornan

**Affiliations:** Department of Urology and Transplantation, Beaumont Hospital, Dublin, 9, Ireland

## Abstract

Metachronous metastasis from renal cell cancer (RCC) after radical surgery can occur in up to one third of patients, most commonly from clear cell RCC. Metastatectomy is a suitable management strategy in oligometastatic disease.

We present the case of a 78-year-old woman who developed haematuria 2 years after left radical nephroureterectomy for a pT3aNx chromophobe RCC (ChRCC). No adjuvant therapy was given and surveillance to date was negative for metastasis.

A large solitary bladder tumour which was resected, and histopathology confirmed intravesical recurrence of the ChRCC. We present this case and discuss intravesical recurrences of renal cancer.

## 1 Introduction

Up to one-third of patients who present with renal cell cancer (RCC) can have metastases at the time of diagnosis, and a further third can develop metastases following curative treatment of the primary.^1^ The burden of metastases is important as those with oligometastatic disease can gain could survival outcomes if the metastases can be excised. Rates of metastases vary with histological subtype of RCC with clear cell subtype most commonly developing metastases Similarly, the distribution of metastases can vary, with the lungs being the most common site of development. Furthermore, the timing of metastases development can be varied, with most metastases developing within 2 years of surgery. We present a case report of a rare entity, a solitary intravesical metastases of a chromophobe RCC almost 3 years after initial nephroureterectomy.

## 2 Case report

KR is a now 78 year old lady who initially presented 3 years ago with an incidentally diagnosed large left renal lesion detected on CT scan, performed as part of a work-up of an alternative medical issue. The patient was asymptomatic without episodes of haematuria or flank pain. Initial diagnostic imaging could not differentiate between RCC and transitional cell carcinoma (TCC). Urinary cytology was negative for high-grade urothelial carcinoma cells. CT scanning did not identify any sites of metastatic disease. Following multidisciplinary meeting (MDM) discussion, a consensus was reached where a radical nephroureterectomy (RNU) should be performed.

RNU was performed with laparoscopic mobilisation of the renal unit and formal open bladder cuff excision and primary bladder closure. Postoperative course was uneventful. The patient did not receive intravesical mitomycin intra- or post-operatively. Final histopathology results revealed a chromophobe renal cell carcinoma and ureteric margins negative for disease, pT3aNxR0. Adjuvant therapy was not advocated, and a schedule of routine CT scanning surveillance was recommended, during which no sites of recurrence were detected.

Approximately 30 months post-surgery, the patient developed significant visible haematuria which required admission to hospital. Rigid cystoscopy was performed and identified a solitary broad-based papillary appearing lesion on the right lateral wall of the bladder. This was completely excised *trans*-urethrally using bipolar resectoscope equipment. Cross-sectional imaging at this time did not identify any other sites of disease.

**Fig. 1:**
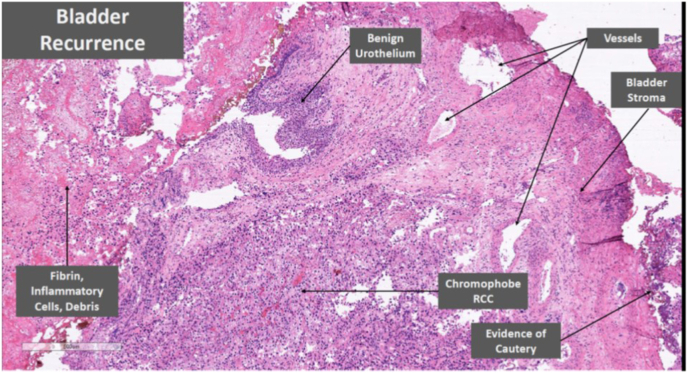
Bladder recurrence of chromophobe RCC.

**Fig. 2:**
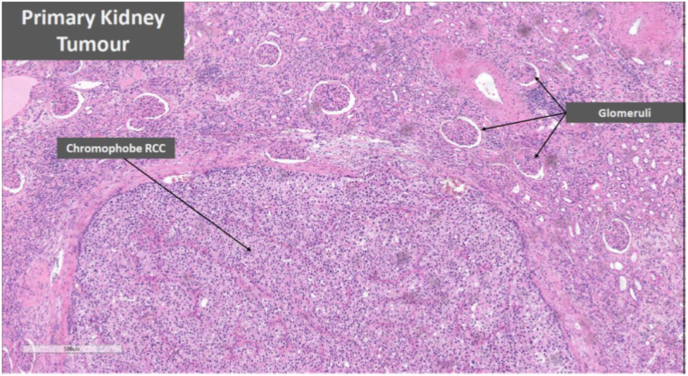
Image of primary renal tumour.

**Fig. 3:**
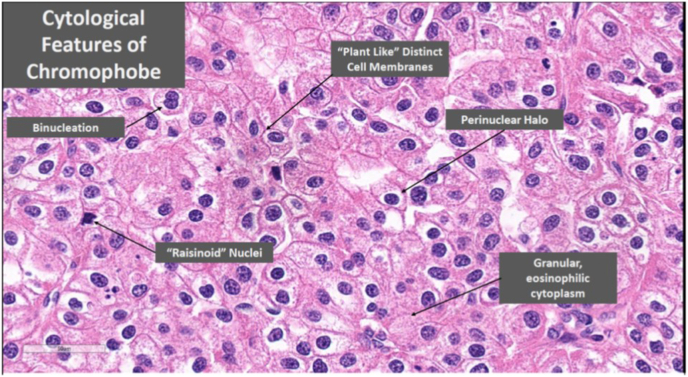
Cytological features of chromophobe RCC noted.

**Fig. 4:**
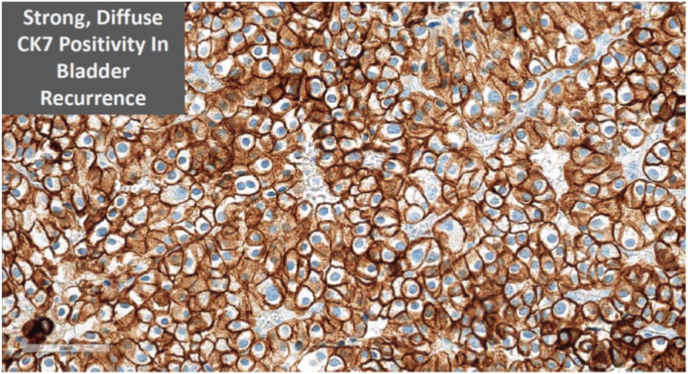
Immunohistochemistry confirmation of chromophobe nature of recurrence.

Histology of this bladder lesion was chromophobe RCC (Fig. 1), consistent with the initial nephrectomy specimen histology (Fig. 2). Specific cytological features of chromophobe RCC are detailed in Fig. 3. Immunohistochemistry confirmed the chromophobe renal cell nature of the recurrence (Fig. 4). Detrusor muscle was included within the specimen and was not involved.

MDM discussion did not recommend further adjuvant treatment given the complete resection status and patients advanced age, and further ongoing surveillance was recommended with no evidence of recurrence within the first 12 months after metastasectomy.

## 3 Discussion

Kidney cancer comprises approximately 3% of all cancers worldwide and have a number of histological subtypes, of which chromophobe renal cell cancer (chRCC) is the least common (approximately 4–6% of all RCCs).^2^ ChRCC arise from the cortical portion of the collecting ducts and in up to 35% of cases, a “central scar” can be identified radiologically. It is not assigned a tumour grade due to inherent nuclear atypia and it usually has the best prognosis of all renal cancer subtypes (88% 10 year cancer-specific survival).^3^

This patient did not undergo a preoperative biopsy of the renal lesion as there was a concern it may have been TCC, which can have additional risks of tumour seeding. It would not have altered course of procedure for this patient significantly as she was deemed suitable for radical surgery which was the appropriate course of action given tumour size. No adjuvant treatment was offered to this patient, in keeping with guidelines of the time.^4^

Rates of metastases at diagnosis with chRCC are low at 4%, as compared with 15% for clear cell carcinoma, and rates of metachronous metastases are also low, comprising only 2.2% of all RCC-related metastases,^3^ with over 58% patients presenting over 1 year after initial diagnosis. The most common sites of metastases were lung and liver. Oligometastatic RCC can be managed with metastatectomy which can be curative if all deposits are excised.^4^

The bladder is an uncommon site of metastases, with one retrospective review identifying metastases to the bladder in only 2.3% of all bladder tumour surgical specimens,^5^ with the most common primary sites being colon, prostate and rectum. Metastases of renal cancer to the bladder is particularly uncommon, with only 65 cases reported in the literature in a series published in 2015.^6^ Metastases to the bladder of chromophobe RCC are rarer still, with only a single other case having been reported previously.^1^

The median time to onset of bladder metastases after initial treatment was 33 months and most patients (72%) presented with haematuria.^6^ 38% patients had other site metastases at time of presentation of bladder metastases, and a further 22% develop other site metastases after treatment of bladder metastases.

Rates of bladder tumour recurrence after nephroureterectomy for upper tract urothelial cell cancer can range from 22 to 47%, significantly higher than the rates reported for RCC.^7^ The antegrade spread of RCC cells via the urinary tract is one proposed mechanism for metastases development within the bladder, alongside haematogenous and lymphatic spread. The rarity of this entity makes it difficult to confirm an exact mechanism. It should be noted that the previously mentioned case series bladder recurrences occurred after both radical nephrectomy, partial nephrectomy and radical nephroureterectomy, suggesting surgical technique may not be relevant.

## 4 Conclusion

We present a case of our 78 year old patient who presented with an uncommon bladder recurrence of her chromophobe renal cell cancer that had been resected via nephroureterectomy some years prior. Complete transurethral resection of the recurrence was performed, and the patient continued regular surveillance without adjuvant therapy.

## Funding sources

None.

## Requirements for authorship

NK: Collected data, prepared manuscript, reviewed manuscript.

LMcL: Reviewed manuscript.

This manuscript has been read and approved by all listed authors. The requirements for authorship have been met and each author believes that this represents an honest account of the work.

## Declaration of competing interest

No conflicts of interest to disclose.
